# Increased sensitivity to TRAIL-induced apoptosis occurs during the adenoma to carcinoma transition of colorectal carcinogenesis

**DOI:** 10.1038/sj.bjc.6602387

**Published:** 2005-02-01

**Authors:** A Hague, D J Hicks, F Hasan, H Smartt, G M Cohen, C Paraskeva, M MacFarlane

**Affiliations:** 1 Department of Oral and Dental Science, University of Bristol, Lower Maudlin Street, Bristol BS1 2LY, UK; 2 Cancer Research Campaign Colorectal Tumour Biology Research Group, Department of Pathology and Microbiology, University of Bristol School of Medical Sciences, University Walk, Bristol BS8 1TD, UK; 3 MRC Toxicology Unit, Hodgkin Building, University of Leicester, PO Box 138, Leicester Road, Leicester LE1 9HN, UK

**Keywords:** TRAIL, colon, adenoma, carcinoma, apoptosis

## Abstract

The death ligand TRAIL (Apo2L) has potential for cancer therapy, since tumour cells are thought to be more sensitive than normal cells. We investigated whether sensitivity to TRAIL increases during the adenoma to carcinoma transition of colorectal carcinogenesis. Under the same culture conditions, we compared the extent of TRAIL-induced apoptosis in four premalignant adenoma and three carcinoma cell lines. Although TRAIL induced some apoptosis in adenoma cultures, the carcinoma cell lines were significantly more sensitive (*P*<0.001). This finding was recapitulated in an *in vitro* model of tumour progression in which conversion of the adenoma cell line AA/C1 to a tumorigenic phenotype was associated with increased TRAIL sensitivity (*P*<0.001). Increased TRAIL sensitivity during colorectal carcinogenesis has been previously attributed to changes in the balance between TRAIL receptors TRAIL-R1 and -R2 and ‘decoy’ receptors TRAIL-R3 and -R4 during malignant progression. To address this, cell surface receptor expression was measured by flow cytometry. In summary, during colorectal carcinogenesis, there is a marked increase in sensitivity to TRAIL-induced apoptosis associated with progression from benign to malignant tumour that could be exploited for colon cancer therapy, but alterations in cell surface TRAIL receptor expression may not be the primary reason for this change.

Tumour necrosis factor-related apoptosis-inducing ligand (TRAIL/Apo2L) mediates rapid apoptosis in cancer cell lines of varied tissue origin (Wiley *et al*, 1995; Pitti *et al*, 1996) and has generated much interest as a potential therapeutic agent due to its apparent differential effects on normal and tumour cells (Wang and El-Deiry, 2003). Tumour necrosis factor-related apoptosis-inducing ligand interacts with four different membrane-bound receptors, two with cytoplasmic death domains, TRAIL-R1 (DR4) and TRAIL-R2 (DR5), and two so-called ‘decoy receptors’ TRAIL-R3 (DcR1) and TRAIL-R4 (DcR2) (Ashkenazi and Dixit, 1998). When TRAIL binds the receptors R1 and R2, the death signal is transmitted through their intracellular protein–protein interacting death domains via cytoplasmic adaptor molecules resulting in an early activation of caspase-8 (Griffith *et al*, 1998; MacFarlane *et al*, 2000a). The activation of the caspase cascade leads, in turn, to cleavage of intracellular substrates such as poly-(ADP-ribose) polymerase (PARP) (Lazebnik *et al*, 1994). However, the death receptors can also transmit signals for cell survival through NF-*κ*B, and via caspase-8 can activate JNK and p38 MAP kinase pathways (Chaudhary *et al*, 1997; Schneider *et al*, 1997; Mühlenbeck *et al*, 1998; MacFarlane *et al*, 2000b). TRAIL-R3 and TRAIL-R4 are unable to transmit the death signal, since TRAIL-R3 does not have a death domain and TRAIL-R4 has a death domain that is substantially deleted (French and Tschopp, 1999). However, in some situations, TRAIL-R4 retains the ability to activate the NF-*κ*B pathway (Degli-Eposti *et al*, 1997). Initially, the differential sensitivity between normal and cancer cells was thought to be due to the presence of decoy receptors on normal cells (Ashkenazi and Dixit, 1998; Green, 1998). More recent studies have failed to find an obvious correlation between decoy receptor expression and the apoptotic response to TRAIL, with levels of TRAIL-R1 and the caspase-8 inhibitory protein c-FLIP being more likely to affect the sensitivity of some cells to TRAIL-induced apoptosis (Kim *et al*, 2000).

Colorectal cancer cells have been reported to be sensitive to TRAIL-induced death (Gliniak and Le, 1999; Lacour *et al*, 2001; Lee *et al*, 2001; Ravi *et al*, 2001). The potential of TRAIL for colorectal cancer treatment is highlighted by the ability of TRAIL to inhibit growth of xenografts of several colon carcinoma cell lines in mouse models and to cooperate with chemotherapeutic agents such as 5-fluorouracil (5-FU), cisplatin, doxorubicin and camptothecin analogue CPT-11 (Ashkenazi *et al*, 1999; Gliniak and Le, 1999; Lacour *et al*, 2001, 2003; Shimoyama *et al*, 2002). In the carcinoma cell line HT29, chemotherapeutic agents, as well as potentiating the mitochondrial amplification of the caspase cascade, enhance recruitment of the adapter protein FADD and caspase-8 to the death-inducing signal complex (DISC) (Lacour *et al*, 2003).

Although Sheikh *et al* (1999) reported overexpression of the decoy receptor TRAIL-R3 mRNA in four out of six colon cancers, with TRAIL-R3 overexpression conferring relative TRAIL resistance, Koornstra *et al* (2003) found no significant alterations in expression of the decoy receptors in colonic tumours compared to normal colonic epithelium *in vivo*. Although loss of heterozygosity of *TRAIL-R2* has been observed in approximately half of colorectal carcinomas, mutations of the two TRAIL receptor genes are rare (Arai *et al*, 1998). The loss of heterozygosity for *TRAIL-R2* is not reflected in the level of protein in the tumours (Koornstra *et al*, 2001), and TRAIL-R1 and TRAIL-R2 proteins are overexpressed in colonic tumours relative to normal colonic mucosa (Koornstra *et al*, 2003). However, in colorectal carcinomas, expression of TRAIL-R1, but not TRAIL-R2, correlates with disease-free survival (Sträter *et al*, 2002a).

Normal colonocytes cultured as intact crypts embedded in collagen have previously been reported to be relatively resistant to TRAIL-induced apoptosis compared to colorectal carcinoma cells (Sträter *et al*, 2002b), but it is not known whether the sensitivity to TRAIL is acquired early or late in colorectal carcinogenesis, since the response of premalignant adenoma cells to TRAIL has never been examined. Koornstra *et al* (2003) showed that, *in vivo*, cytoplasmic TRAIL-R1 and -R2 expression was higher in both adenomas and carcinomas compared to normal colonic epithelium; however, there were no further changes in expression of either TRAIL receptors or decoy receptors between adenomas and carcinomas. From these results, it might be assumed that the sensitivity to TRAIL is acquired early in colorectal tumorigenesis during the formation of the adenoma, and that this is due to the changes in TRAIL receptor expression. However, the cell surface expression of TRAIL receptors in adenoma cells has not been examined, and this could have an important bearing on the relative TRAIL sensitivity of colorectal epithelial cells during tumour progression. In this study, we addressed whether TRAIL sensitivity increases in association with the adenoma to carcinoma transition by comparing the response of four adenoma cell lines and three carcinoma cell lines to TRAIL under the same culture conditions. This is important because Larribere *et al* (2004) have shown that the response to TRAIL is dependent on culture conditions and that growth factor signalling through phosphatidyl-inositol-3 kinase and its downstream target, Akt, attenuates TRAIL-induced apoptosis of normal human melanocytes. In addition, we used a model of tumour progression in which an adenoma cell line had been transformed to a malignant phenotype *in vitro* (Williams *et al*, 1990); this allowed us to measure directly whether TRAIL sensitivity increased with acquisition of malignancy, again under the same culture conditions. To assess whether the relative abundance of TRAIL receptors on the cell surface accounted for differences in sensitivity to TRAIL-induced apoptosis, we measured cell surface expression of TRAIL-R1, -R2, -R3 and -R4 by flow cytometry.

## MATERIALS AND METHODS

### Cell lines and culture conditions

The adenoma cell lines used were AA/C1, RG/C2, AN/C1 and RR/C1. These cells were capable of growth after trypsinisation to single cells and are derivatives of the cell lines PC/AA (Paraskeva *et al*, 1984), S/RG (Paraskeva *et al*, 1989), S/AN (Paraskeva *et al*, 1989) and S/RR (Williams *et al*, 1992). The carcinoma cell lines used were HT29 (Fogh and Trempe, 1975), SW620, derived from a lymph node metastasis (Leibovitz *et al*, 1976), and KS (Hague *et al*, 1997), which was derived in our laboratory using the same techniques as for the adenoma cultures. Whereas the four adenoma cell lines were anchorage-dependent and did not form tumours in nude mice (Williams *et al*, 1990; Hague *et al*, 1997), the carcinoma cell lines were anchorage-independent and tumorigenic after subcutaneous injection in nude mice. The AA/C1/SB/10 cell line is an *in vitro*-transformed variant of the AA/C1 adenoma cell line that is anchorage-independent and forms tumours in nude mice (Williams *et al*, 1990).

All of the cell lines used in this study were grown in Dulbecco's modified Eagle's medium (DMEM) (Gibco) containing 20% fetal bovine serum (Gibco, batch selected), glutamine (2 mM), insulin (0.2 u ml^−1^), hydrocortisone sodium succinate (1 *μ*g ml^−1^), penicillin (100 u ml^−1^) and streptomycin (100 *μ*g ml^−1^). Cells were grown in T25 flasks and were routinely passaged using trypsin (0.1%) (Difco, West Molesey, Surrey, UK)/EDTA (0.1%) (AnalaR, BDH).

### Preparation of TRAIL and treatment of cells

The TRAIL preparation used was recombinant soluble TRAIL with N-terminal T7 and His_6_ tags obtained by nickel affinity purification as previously described (MacFarlane *et al*, 1997). For dose–response experiments, a stock solution of TRAIL (1 mg ml^−1^) was prepared in EDTA (150 mM) and diluted in medium to give final concentrations of 0.25, 0.5 and 1.0 *μ*g ml^−1^. Duplicate or triplicate flasks were treated with each TRAIL concentration or EDTA only. Control cultures were treated with the vehicle only.

### Identification of apoptosis

As previously demonstrated in these cells (Hague *et al*, 1993), apoptosis results in rapid detachment from the flask and the apoptotic cells float in the medium. Floating cells were collected at the end of the treatment time and adherent cells were harvested by trypsinisation. Adherent and floating cells were quantified using a counting chamber. The extent of apoptotic cell shedding was calculated as the number of floating cells per flask divided by the total cell yield (number of adherent plus floating cells). Adherent and floating cells were examined separately for apoptotic morphology by acridine orange staining as described previously (Hague *et al*, 1993). Unfixed cells were stained with 5 *μ*g ml^−1^ acridine orange in PBS for 10 min and were then viewed by fluorescence microscopy. For each experiment, the cells shed into the medium were confirmed to be morphologically apoptotic with condensed chromatin. At least 150 cells were scored per sample. To further demonstrate that TRAIL induced apoptotic death, adherent and floating cells were examined separately for DNA laddering as described previously (Hague *et al*, 1993). Cleavage of PARP was detected by Western blot analysis.

### SDS–PAGE–Western blotting

Western blotting was carried out used standard techniques and antibody binding was detected using an enhanced chemiluminescence (ECL) detection kit (Amersham, Buckinghamshire, UK) following the manufacturer's protocol. To detect BID, two antibodies were used: a polyclonal BID antibody (1 : 200) kindly provided by Dr Xiadong Wang (Howard Hughes Medical Institute, Texas) and a polyclonal BID antibody (AF860) (1 : 1000) from R&D systems. c-FLIP rabbit polyclonal antibody was a gift from Dr D Nicholson (Merck Frosst, Canada) and was used at 1 : 1000. Results were confirmed using a mouse monoclonal antibody, NF-6 (Alexis Corporation) at 1 : 1000. XIAP was detected with a mouse monoclonal antibody used at 1 : 500 (BD Transduction Laboratories). A mouse monoclonal *α*-tubulin antibody (Sigma, Poole, Dorset, UK) or a mouse monoclonal *α*-GAPDH antibody (Advanced Immunochemical Inc., CA, USA) was used to assess equal loading of the gels, both at 1 : 2000. Secondary antibodies were anti-mouse or anti-rabbit horseradish peroxidase-conjugated antibodies (Sigma).

### Flow cytometric analysis of cell surface death receptor expression

TRAIL-R1, -R2, -R3 and -R4 receptor antibodies (clones M272, M413, M430 and M444, respectively) were kind gifts from Immunex Corp. (Seattle, WA, USA) and have been described previously (Griffith *et al*, 1999). These antibodies have been validated for flow cytometric measurement of surface TRAIL expression (Zhang *et al*, 2000; MacFarlane *et al*, 2002). A total of 10^6^ trypsinised colon cells (live) were incubated in blocking buffer (PBS containing 10% normal goat serum) for 30 min on ice to block nonspecific binding. They were then incubated for 1 h with anti-TRAIL receptor antibodies (diluted 1 : 50) at 4°C. After two washes in PBS and centrifugation for 5 min at 400 **g**, the cells were resuspended in blocking buffer containing goat anti-mouse FITC-conjugated antibody (F(ab)_2_ fragment) (DAKO) (1 : 20 dilution) and incubated for 1 h at 4°C. The cells were then washed twice in cold PBS prior to analysis by flow cytometry (FACScan, Becton Dickinson). Dead cells were excluded by costaining the cells with propidium iodide. The propidium iodide-positive cells could then be gated out.

### Statistical analysis

The response of adenomas and carcinomas was compared by a two-way analysis of variance of TRAIL-induced apoptosis (malignant potential × TRAIL concentration). For the comparison of TRAIL sensitivity between the parental adenoma cell line AA/C1 and the transformed adenoma cell line AA/C1/SB/10, a two-way analysis of variance was conducted (cell line × TRAIL concentration). All analyses were conducted using SPSS 10 for Windows software.

## RESULTS

### Colorectal cancer cells are more sensitive to TRAIL-induced apoptosis than premalignant adenoma cells

**Figure 1:**
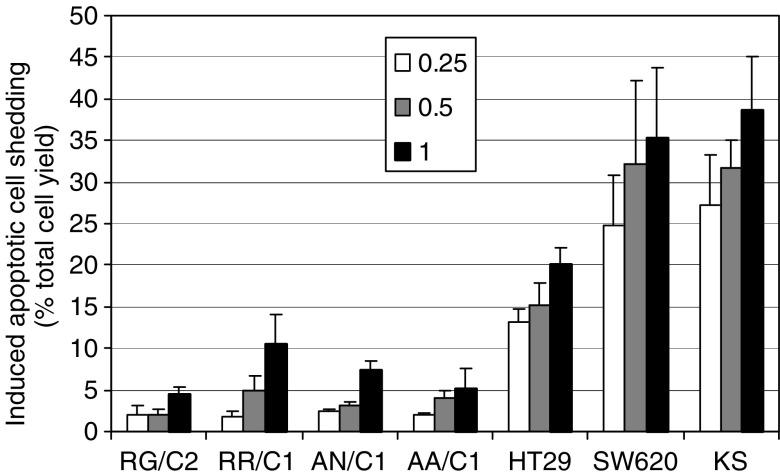
Induction of apoptotic cell shedding by TRAIL in four colorectal adenoma cell lines (RG/C2, RR/C1, AN/C1 and AA/C1) and in three carcinoma cell lines (HT29, SW620 and KS). Cells were treated in exponential growth with either vehicle only or 0.25, 0.5 or 1.0 *μ*g ml^−1^ TRAIL for 16 h. The floating cells and adherent cells were counted separately. Floating cells were confirmed as apoptotic by acridine orange staining for each experiment. The extent of apoptosis was calculated as the proportion of the total cells in the flask that were floating in the medium. For each experiment, TRAIL-induced apoptosis is shown as the percentage of apoptotic cells shed into the medium over and above that in the control cultures. Data are means±s.e.m. of at least three experiments conducted either in duplicate or triplicate. The percentages of floating apoptotic cells in the control cultures were as follows: 12.12% for RG/C2, 3.66% for RR/C1, 1.85% for AN/C1, 3.55% for AA/C1, 1.01% for HT29, 3.56% for SW620 and 3.47% for KS. The mean induced apoptotic cell shedding was compared between adenoma-derived cell lines and carcinoma-derived cell lines using a two-way analysis of variance. The difference was highly statistically significant (*P*<0.001).

In this study, we addressed whether there is differential sensitivity to TRAIL-induced apoptosis between malignant and premalignant colorectal epithelial cells. Initially, the apoptotic response of four premalignant, nontumorigenic adenoma cell lines (RG/C2, RR/C1, AN/C1 and AA/C1) was compared with that of three carcinoma cell lines (HT29, SW620 and KS) by conducting dose–response experiments for each cell line. In order to determine the extent of resistance of the adenoma cells, relatively high concentrations of TRAIL were used (0.25–1 *μ*g ml^−1^). As in our previous studies quantifying apoptosis in colon cells, we used cell shedding as an assessment of the extent of apoptosis (Hague *et al*, 1993). The cells shed into the medium in response to TRAIL treatment were confirmed to be apoptotic by acridine orange staining and examination of the floating cells by fluorescent microscopy for condensed chromatin, PARP cleavage to its 85 kDa form and DNA laddering (data not shown). Treatment with TRAIL did not induce necrosis in any of the cell lines even at concentrations up to 2 *μ*g ml^−1^. Measurements of the extent of apoptotic cell shedding after a 16 h treatment demonstrated that, although TRAIL induced apoptosis in the adenoma cell cultures, the carcinoma cell lines were markedly more sensitive to TRAIL than the adenoma cell lines (Figure 1). When extensive shedding was obtained over the 16 h treatment period, this was reflected in reduced adherent cell yields (data not shown). The three carcinoma cell lines were markedly more sensitive than the four adenoma cell lines, a difference that was highly statistically significant (*P*<0.001).

**Figure 2:**
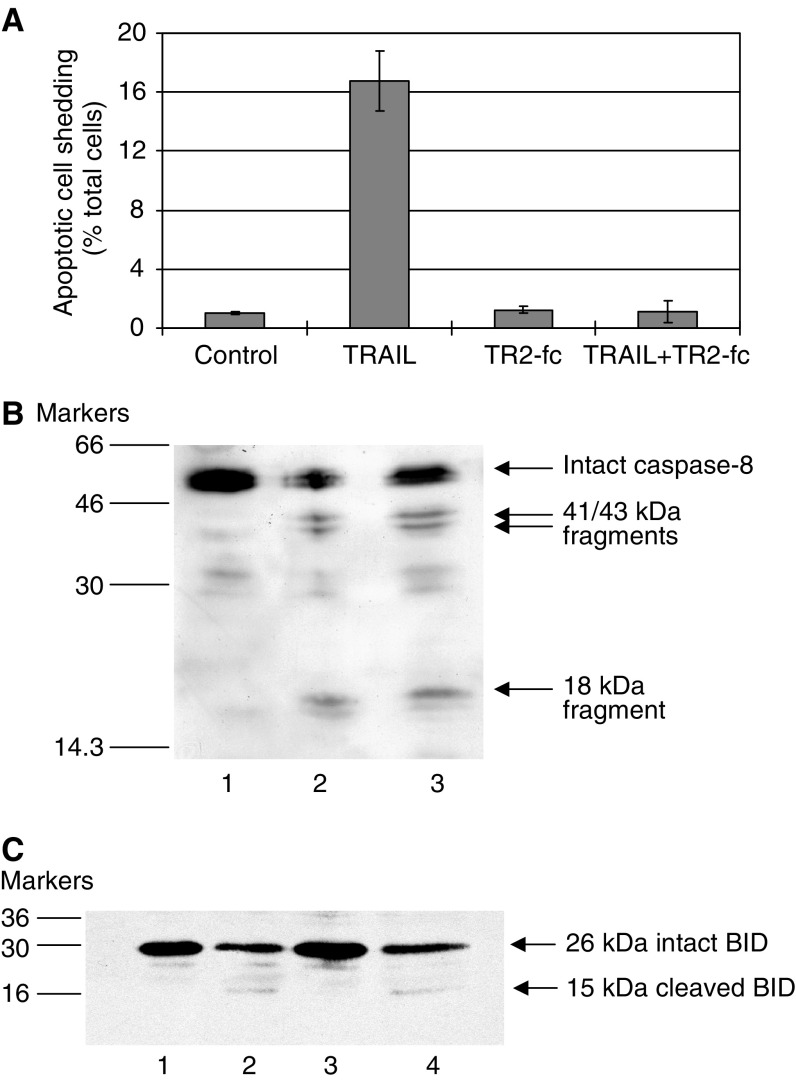
(**A**) Apoptosis induced by TRAIL is receptor-mediated and involves cleavage of caspase-8 and BID. Apoptosis induced by TRAIL in the HT29 cell line is blocked using TRAIL R2-Fc chimeric protein (R and D systems, Abingdon, UK) to compete with cellular TRAIL receptors for the death ligand. Apoptotic cell shedding induced by 0.25 *μ*g ml^−1^ TRAIL was completely blocked by 300 *μ*g ml^−1^ TRAIL-R2-Fc. (**B**) Caspase-8 is cleaved in HT29 cells in response to TRAIL treatment (16 h). Lane 1: adherent control cells; lane 2: adherent cells treated with 0.125 *μ*g ml^−1^ TRAIL; lane 3: adherent cells treated with 0.25*μ*g/ml TRAIL. (**C**) BID is cleaved in HT29 cells in response to 16 h TRAIL treatment. Lane 1: control adherent cells; lane 2: TRAIL-treated adherent cells (0.25 *μ*g ml^−1^); lane 3: control adherent and floating cells pooled; lane 4: TRAIL-treated adherent and floating cells pooled.

To confirm that TRAIL-mediated apoptosis was due to receptor ligation and not due to nonspecific toxicity of TRAIL, a TRAIL-R2-Fc chimeric protein, comprised of the TRAIL-R2 extracellular domain fused to human IgG_1_ Fc, was used as a soluble receptor and blocking reagent. TRAIL-R2-Fc completely blocked TRAIL-induced apoptotic cell shedding in the carcinoma cell line HT29, confirming that death receptor-mediated apoptosis was responsible for the cell shedding induced by TRAIL treatment (Figure 2A). Consistent with this, TRAIL treatment resulted in activation of caspase-8, with cleavage of the proform of 55 kDa to 43, 41 and 18 kDa fragments (Figure 2B) together with cleavage of the 26 kDa proform of BID to yield its 15 kDa fragment (Figure 2C). Cleavage of caspase-8 and BID was accompanied by reduced levels of the full-length forms (Figure 2B and C).

### The *in vitro*-transformed adenoma cell line AA/C1/SB/10 is more sensitive to TRAIL-induced apoptosis than the parental adenoma cell line AA/C1

**Figure 3:**
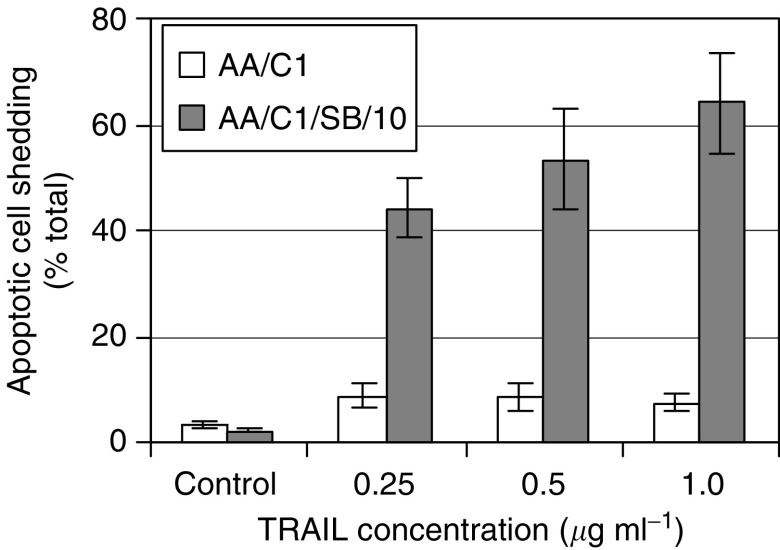
The *in vitro*-transformed, tumorigenic AA/C1/SB/10 cell line is more sensitive to TRAIL-induced apoptosis than the adenoma cell line AA/C1 from which it was derived. Dose response of the adenoma cell line AA/C1 and the *in vitro*-transformed adenoma cell line AA/C1/SB/10 showing the extent of apoptotic cell shedding after a 16 h treatment with 0.25, 0.5 and 1 *μ*g ml^−1^ TRAIL. Results shown are means±s.e.m. of three experiments performed in triplicate. Comparing the percentage of apoptotic cell shedding between AA/C1 and AA/C1/SB/10 by a two-way analysis of variance revealed a significantly greater extent of apoptosis in AA/C1/SB/10 (*P*<0.001).

Since adenoma cells appeared to be more resistant to TRAIL than carcinoma cells, experiments were conducted to determine more directly whether TRAIL sensitivity increased in association with malignant progression by comparing the sensitivity of AA/C1, an anchorage-dependent, nontumorigenic, adenoma-derived cell line, and AA/C1/SB/10, an anchorage-independent, tumorigenic derivative of the AA/C1 cell line (Williams *et al*, 1990). In these experiments, cell shedding was confirmed to be due to apoptosis by acridine orange staining for apoptotic morphology and by detection of PARP cleavage (data not shown). Although the transformed AA/C1/SB/10 cell line had lower levels of spontaneous apoptosis than the parental AA/C1 cell line, dose–response experiments demonstrated that AA/C1/SB/10 was more sensitive to TRAIL-induced apoptosis than AA/C1 (*P*<0.001) (Figure 3). The AA/C1 cells remained relatively resistant with increasing concentrations of TRAIL (0.25, 0.5 and 1 *μ*g ml^−1^). Even 2 *μ*g ml^−1^ TRAIL did not induce as much apoptotic cell shedding in AA/C1 as that induced by 0.25 *μ*g ml^−1^ in AA/C1/SB/10 (data not shown). Therefore, the differential sensitivity between the two cell lines was evident even at high TRAIL concentrations. Furthermore, the differential sensitivity was maintained even after 96 h exposure to TRAIL (data not shown). It can be concluded that AA/C1 became more sensitive to TRAIL-induced apoptosis during malignant transformation *in vitro*.

### Cell surface receptor expression profiles of adenoma and carcinoma cells cannot account for their differential sensitivity to TRAIL

**Figure 4:**
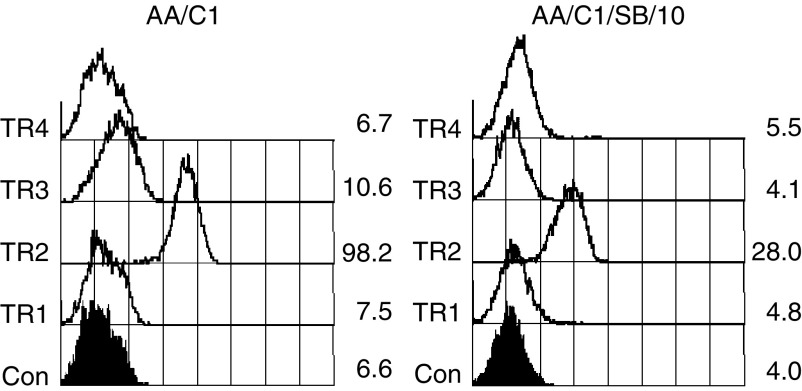
Cell surface receptor expression in the adenoma cell line AA/C1 (premalignant) and the *in vitro*-transformed derivative AA/C1/SB/10 (malignant). Cell surface expression of the four TRAIL receptors was detected by single-colour flow cytometric analysis of live (propidium iodide-excluding) cells stained with monoclonal antibodies and detected using a FITC-conjugated anti-mouse secondary antibody (See legend to Table 1, values shown correspond to Mean Fluorescence Intensity). Control cells were incubated with secondary antibody only. The experiment was repeated with similar results.

Since it has been reported that there are no differences in total TRAIL receptor expression between colorectal adenomas and carcinomas (Koornstra *et al*, 2003), we addressed whether the differential sensitivity between nonmalignant and malignant colonic epithelial cells could be explained by relative cell surface expression of TRAIL receptors. To do this, flow cytometric analysis of antibody-labelled nonfixed cells was performed. The antibodies used to detect TRAIL receptors have been well characterised for efficient receptor binding by Griffith *et al* (1999) and for detection of cell surface TRAIL receptors by flow cytometry (Zhang *et al*, 2000; MacFarlane *et al*, 2002). TRAIL-R1 levels were low and similar in adenoma and carcinoma cell lines (Table 1). TRAIL-R2 was expressed in all of the eight cell lines and was the most abundant of the four receptors. AA/C1, which was relatively resistant to TRAIL, had the highest levels of TRAIL-R2, whereas KS, which was sensitive to TRAIL-induced apoptosis, had low levels. TRAIL-R3 was expressed in the adenoma cell lines AN/C1 and AA/C1, and in the transformed adenoma cell line AA/C1/SB/10, but interestingly, the other five colonic tumour cell lines did not express cell surface TRAIL-R3. Similarly, only three cell lines expressed the TRAIL-R4 receptor (at low levels): the carcinoma cell line HT29, the adenoma cell line AN/C1 and the transformed adenoma cell line AA/C1/SB/10. The expression pattern of the TRAIL receptors did not correlate with sensitivity to TRAIL or the malignant potential of the cell lines. Similarly, the receptor profiles of the AA/C1 cell line and the transformed adenoma cell line AA/C1/SB/10 did not explain why the transformed derivative was more sensitive to TRAIL-induced apoptosis (Figure 4 and Table 1).

### The relative resistance of the adenoma cell lines to TRAIL-induced apoptosis cannot be accounted for by higher expression of XIAP or c-FLIP

**Figure 5:**
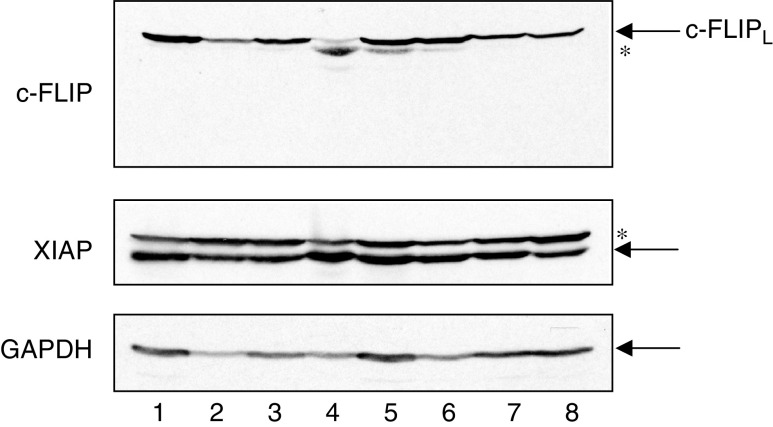
The relative resistance of the adenoma cell lines to TRAIL-induced apoptosis cannot be accounted for by higher expression of XIAP or c-FLIP. c-FLIP and XIAP expression in the four adenoma cell lines, the transformed adenoma cell line and the three carcinoma cell lines is shown. Lane 1: RG/C2; lane 2: RR/C1; lane 3: AN/C1; lane 4: AA/C1; lane 5: AA/C1/SB/10; lane 6: HT29; lane 7: SW620; lane 8: KS. Bands corresponding to the full-length proteins are arrowed. The asterisks denote nonspecific bands. Since XIAP is of similar size to *α*-tubulin, GAPDH was used as a loading control for this blot.

Given the lack of correlation between malignant potential and TRAIL receptor expression, we further investigated the relative resistance of adenoma cell lines to TRAIL by examining intracellular components of the death receptor signalling pathway that have previously been shown to modulate TRAIL sensitivity. c-FLIP, an inhibitor of caspase-8 activation, and XIAP, an inhibitor of active caspases-3 and -9, were expressed in all of the cell lines. c-FLIP_L_ levels were markedly higher in the transformed adenoma cell line AA/C1/SB/10 than in the parental adenoma cell line AA/C1 (Figure 5, top panel, compare lanes 4 and 5). Thus, c-FLIP levels did not appear to correlate with TRAIL sensitivity. Although c-FLIP_S_ serves to inhibit TRAIL-induced apoptosis in some cell types (Bin *et al*, 2002), none of the colorectal tumour cell lines expressed c-FLIP_S_. XIAP levels also did not appear to correlate with TRAIL sensitivity. Although XIAP levels were slightly higher in the parental adenoma cell line AA/C1 than in AA/C1/SB/10, the other cell lines had similar expression levels of XIAP (Figure 5, middle panel).

## DISCUSSION

Interest in TRAIL as a potential chemotherapeutic agent stems from observations suggesting that it may selectively induce apoptosis in cancer cells while sparing normal cells. Increased sensitivity to TRAIL during carcinogenesis is not a feature of all tissues; for example, comparisons of the response of normal and malignant ovarian cells to TRAIL have shown normal cells to be sensitive to TRAIL whereas cells from different ovarian carcinomas varied in their responses (Lane *et al*, 2004). Normal colonocytes have previously been reported to be relatively resistant to TRAIL-induced apoptosis compared to colorectal carcinoma cells: normal human colonic epithelial cells did not undergo apoptosis in response to 0.1 *μ*g ml^−1^ TRAIL, but COLO205 colon carcinoma cells were sensitive to this concentration (Sträter *et al*, 2002b). Koornstra *et al* (2003) reported that colonic tumours overexpressed the TRAIL receptors TRAIL-R1 and TRAIL-R2 *in vivo* relative to normal mucosa, but that overall expression of TRAIL receptors was not significantly different between adenomas and carcinomas. On the basis of these observations, one might predict that the acquisition of TRAIL sensitivity would occur early in colorectal carcinogenesis and that adenoma cells would be as sensitive to TRAIL-induced apoptosis as carcinoma cells. However, it was not known whether there was a difference in TRAIL sensitivity between adenoma and carcinoma cells and whether TRAIL receptor expression on the cell surface, rather than intracellular receptor pools, would correlate with sensitivity to TRAIL-induced apoptosis. We addressed this hypothesis by comparing the extent of apoptosis induced by TRAIL in nonmalignant and malignant colorectal tumour cell lines. We initially questioned whether TRAIL was able to induce apoptosis of human colorectal adenoma cells, since this has not been reported before. The adenoma-derived cell lines did exhibit some apoptosis in response to TRAIL; however, all four adenoma cell lines, AA/C1, AN/C1, RR/C1 and RG/C2, were markedly more resistant than the three carcinoma cell lines HT29, SW620 and KS. The differential sensitivity between the adenoma and carcinoma cell lines was highly statistically significant (*P*<0.001). Furthermore, we used an *in vitro* model of colorectal carcinogenesis to demonstrate that the adenoma cell line AA/C1 became more sensitive to TRAIL after *in vitro* transformation to a malignant phenotype (*P*<0.001). Our studies provide direct evidence for increasing TRAIL sensitivity during colorectal carcinogenesis during the adenoma to carcinoma transition. They also show that the increased sensitivity to TRAIL can be recapitulated *in vitro*, and therefore *in vivo* selection pressures are not necessarily required for the acquisition of TRAIL sensitivity. The increased TRAIL sensitivity may relate to increasing growth rate or growth autonomy, or to the attainment of anchorage independence.

In normal colonic epithelium, apoptosis is primarily at the crypt lumen (Brodie *et al*, 2004), and for the most part at the top of the crypts (Hall *et al*, 1994). In early adenomas, there is apoptosis close to the basement membrane, particularly at crypt branching points (Brodie *et al*, 2004), whereas crypt lumen apoptosis is a feature of larger adenomas, high-grade dysplasia and adenocarcinomas. It is unknown to what extent TRAIL responsiveness contributes to the changing patterns of apoptosis in human colorectal tumours. However, the contribution of TRAIL-R2 to colorectal tumour growth and apoptosis sensitivity has recently been demonstrated by Wang and El-Deiry (2004) using stable human colon cancer cell lines in which the function of TRAIL-R2 was ablated using inducible RNA interference in a mouse xenograft model. Inducible silencing of TRAIL-R2 *in vivo* accelerated growth of bioluminescent tumour xenografts and conferred resistance to the chemotherapeutic agent 5-FU. Sträter *et al* (2002a) found that in normal colonic epithelium, TRAIL and TRAIL-R2 were expressed primarily in the surface epithelium, whereas TRAIL-R1 and TRAIL-R4 were detected all along the crypt axis. In adenomas, this expression pattern was mostly retained, although some adenomas also expressed abnormally high levels of TRAIL-R3. In carcinomas, the expression of TRAIL and TRAIL receptors was much more variable, but TRAIL-R1 expression was significantly associated with disease-free survival. Koornstra *et al* (2003) also reported overall expression of TRAIL receptors, which have a large cytoplasmic component, to be similar in adenoma and carcinoma cells; therefore, immunohistochemical studies have not revealed a mechanism for the differences in TRAIL sensitivity between the adenoma and carcinoma cells. We therefore examined the cell surface expression of the four TRAIL receptors by flow cytometry. The importance of examining cell surface expression of TRAIL receptors has been highlighted recently by Jin *et al* (2004), who showed that TRAIL-resistant variants of the colon carcinoma cell line SW480 had reduced cell surface expression of TRAIL-R1 compared to the parental cell line, although total protein expression remained unchanged. However, it is interesting that in our study the cell surface expression of the four TRAIL receptors did not correlate with the increased sensitivity to TRAIL that is associated with malignancy. Importantly, this indicates that the increased sensitivity to TRAIL may instead be due to alterations in intracellular signalling. In this regard, it is worth noting that we found no correlation between the expression of c-FLIP or XIAP, or of Bcl-2 family members Bcl-2, Bcl-xL, Bax or Bak to explain the differential sensitivity (data not shown). Furthermore, TRAIL-induced NF-*κ*B activity did not correlate with malignant potential or sensitivity to TRAIL-induced apoptosis (data not shown).

In summary, using an *in vitro* model of colorectal tumour progression, we have demonstrated for the first time that colorectal adenoma cells are more resistant to TRAIL-induced apoptosis than carcinoma cells and that TRAIL sensitivity increases during the conversion of an adenoma cell line to a malignant phenotype. Importantly, since the adenoma cultures are grown under the same culture conditions as the carcinoma cultures, this cannot be attributed to differences in survival factors or medium composition. The results are important for the potential application of TRAIL as a treatment for colorectal cancer because of the current focus on the mechanisms of TRAIL resistance in colorectal tumours – it will be important to define the ‘window’ of TRAIL sensitivity. Although the mechanism for the differential sensitivity of benign and malignant cells to TRAIL remains elusive, our data indicate that death receptor expression alone cannot explain why cells become sensitive to TRAIL on acquisition of the malignant phenotype. Therefore, in addition to defining mechanisms by which resistant variants may subsequently arise after prolonged TRAIL treatment, further investigations are also needed to define the mechanisms by which colorectal carcinoma cells become sensitive to TRAIL and what selective advantage of tumour cells has given rise to this sensitivity. Together, these investigations will assist in the optimisation of TRAIL for therapeutic use.

## Figures and Tables

**Table 1:** Flow cytometric analysis of TRAIL receptor expression

	**Adenoma cell lines**				**Transformed adenoma**	**Carcinoma cell lines**		
	**RG/C2**	**RR/C1**	**AN/C1**	**AA/C1**	**AA/C1/SB/10**	**HT29**	**SW620**	**KS**
CON^a^	3.72	5.48	7.22	6.09	3.79	3.37	3.79	2.37
NEG^b^	4.28	5.95	7.26	6.61	3.98	3.65	4.05	2.55
								
TRAIL-R1	5.50	7.49	8.70	7.55	4.84	5.19	5.84	2.81
TRAIL-R2	13.75	25.69	27.14	98.18	27.97	17.01	24.67	4.52
TRAIL-R3	4.10	5.90	9.39	10.60	4.10	3.92	3.91	2.49
TRAIL-R4	4.31	5.80	8.02	6.67	5.54	4.38	4.20	2.48

Values are the mean fluorescent intensity of live (propidium iodide-excluding) cells stained with monoclonal antibodies and detected using an FITC-conjugated mouse secondary antibody.

a Cells incubated without primary or secondary antibody.

b Cells incubated with secondary antibody only.
